# SARS-CoV-2 Variants of Concern and Clinical Severity in the Mexican Pediatric Population

**DOI:** 10.3390/idr15050053

**Published:** 2023-09-11

**Authors:** Anahí Maldonado-Cabrera, Jesus Alejandro Colin-Vilchis, Ubydul Haque, Carlos Velazquez, Andrea Socorro Alvarez Villaseñor, Luis Eduardo Magdaleno-Márquez, Carlos Iván Calleros-Muñoz, Karen Fernanda Figueroa-Enríquez, Aracely Angulo-Molina, Ana Lucía Gallego-Hernández

**Affiliations:** 1 Department of Chemical Biological Sciences, University of Sonora, Hermosillo 83000, Mexico; a201203057@unison.mx (A.M.-C.); velaz2@unison.mx (C.V.); 2 Department of Epidemiology, Family Medicine Unit No. 37, Mexican Social Security Institute (IMSS), Hermosillo 83260, Mexico; 3 Department of Medicine, Autonomous University of the Mexico State, Toluca 50000, Mexico; jcolinv859@alumno.uaemex.mx; 4 Rutgers Global Health Institute, New Brunswick, NJ 08901, USA; uhaque@globalhealth.rutgers.edu; 5 Department of Biostatistics and Epidemiology, School of Public Health, Rutgers University, Piscataway, NJ 08854, USA; 6 Coordination of Medical Health Research, Mexican Social Security Institute (IMSS), La Paz 23920, Mexico; andrea.alvarez@imss.gob.mx; 7 University Center for Biological and Agricultural Sciences, University of Guadalajara, Zapopan 44100, Mexico; eduardo.magdaleno@alumnos.udg.mx; 8 Department of Agriculture, University of Sonora, Hermosillo 83323, Mexico; a219213704@unison.mx (C.I.C.-M.); a219218338@unison.mx (K.F.F.-E.); 9 School of Life Sciences, University of Applied Sciences and Arts Northwestern Switzerland, 4132 Muttenz, Switzerland

**Keywords:** children, COVID-19, severity, hospitalization, mortality, pediatrics

## Abstract

The emergence of severe acute respiratory syndrome coronavirus-2 (SARS-CoV-2) variants of concern (VOCs) presents global heterogeneity, and their relative effect on pediatric severity is still limited. In this study, we associate VOCs with pediatric clinical severity outcomes in Mexico. Bioinformatics methods were used to characterize VOCs and single amino acid (aa) mutations in 75,348 SARS-CoV-2 genetic sequences from February 2020 to October 2022. High-predominance VOCs groups were calculated and subsequently associated with 372,989 COVID-19 clinical pediatric outcomes. We identified 21 high-frequency mutations related to Omicron lineages with an increased prevalence in pediatric sequences compared to adults. Alpha and the other lineages had a significant increase in case fatality rate (CFR), intensive critical unit (ICU) admission, and automated mechanical ventilation (AMV). Furthermore, a logistic model with age-adjusted variables estimated an increased risk of hospitalization, ICU/AMV, and death in Gamma and Alpha, in contrast to the other lineages. We found that, regardless of the VOCs lineage, infant patients presented the worst severity prognoses. Our findings improve the understanding of the impact of VOCs on pediatric patients across time, regions, and clinical outcomes. Enhanced understanding of the pediatric severity for VOCs would enable the development and improvement of public health strategies worldwide.

## 1. Introduction

Genetic mutations in the severe acute respiratory syndrome-related coronavirus (SARS-CoV-2) have changed the clinical outcomes of COVID-19 patients. However, there is still limited information on how SARS-CoV-2 genetic variations affect pediatric severity. The relatively low number of reported pediatric cases is the result of a combination of factors, like low medical attention (due to mild and asymptomatic cases), isolation, and high recovery rates during COVID-19 disease [1].

The SARS-CoV-2 virus belongs to the Coronaviridae family of the Nidovirales order, and harbors a linear single-stranded positive RNA genome (+ssRNA). The genome size of 29.9 kb encodes 27 proteins, which include 15 non-structural proteins (NSP), 8 accessories, and 4 major structural proteins [2]. Structural proteins correspond to the spike glycoprotein (S protein), membrane protein (M protein), envelope (E protein), and nucleocapside (N protein) (Figure S1). Preliminary estimates of the evolutionary rate of SARS-CoV-2 are close to ~6 × 10^−4^ nucleotides/genome/year, similar to other RNA virus genomes [3]. This pro-mutational state has led to significant RNA structural changes over time. The World Health Organization (WHO) has characterized SARS-CoV-2 variants’ with an increased risk to global public health as Variants of Concern (VOCs) [4]. The introduced VOCs lineages are Alpha (B.1.1.7), Beta (B.1.351), Gamma (P.1), Delta (B.1.617.2), and Omicron (B.1.1.529, BA.1 to BA.5) [5]. These VOCs have shown an increase in clinical severity and changes in transmission, diagnostic techniques, therapeutics, and vaccine effectiveness [6]. 

SARS-CoV-2 variants have been identified and tracked by multiple surveillance genomic processes [7,8]. Thanks to the worldwide effort of scientists, governments, and the global initiative on sharing all influenza data (GISAID), genetic sequences were made publicly available to the research community [9]. The WHO and the Center for Disease Prevention (CDC) define SARS-CoV-2 variants periods of high predominance through mathematical models [10]. These calculations are useful to assess changes in the dynamics, transmission speed, and clinical evolution of COVID-19, as well as for planning public health actions. It has been described how these genetic variants can modify the transmission dynamic over geographical regions, with certain VOCs emerging and disappearing while others endure [11,12]. 

COVID-19 pediatric patients commonly have a mild course in most cases, with low hospitalization and mortality rates [13]. Some of the reported pediatric physiological advantages are based on immune system mechanisms. These factors include a stronger innate immune response, a lower hyperinflammation response, and lower proinflammatory cytokine responses, among others [14]. However, the COVID-19 complications that can affect pediatric populations include, but are not limited to, respiratory, metabolic, sepsis, and nosocomial infections, and systemic inflammatory syndrome [15].

In Mexico, a country of geographical contrasts, multiple health factors influence COVID-19 clinical outcomes in younger populations [16]. These factors include national vaccination coverage, socioeconomic variables, and access to health services [17]. Additionally, Mexico shares a border with the United States of America (USA), creating a social migration determinant where thousands of individuals travel back and forth to the USA from different Latin American countries [18]. These socioeconomic factors have established a unique geographical situation where different infectious diseases of health concern can converge [19].

In Mexico, the first case of COVID-19 was introduced in February 2020 [20]. Afterward, in 2021, the VOCs’ infiltration presented a rapid dispersion affecting all age groups. COVID-19 morbidity, hospitalization, and death data were processed throughout the pandemic on open-access platforms [21]. Similarly, SARS-CoV-2 genetic sequences from all over the country were collected, processed, and deposited in the GISAID repository. However, in Mexican pediatric patients, clinical severity indicators such as hospitalization, mortality, and ICU/AMV based on the SARS-CoV-2 genetic mutations’ prevalence remain poorly investigated. 

Therefore, in this study we assessed the clinical severity of Mexican pediatric patients during periods of VOCs high predominance. This evaluation is relevant to identify the most virulent VOCs, address specific therapies and vaccines, and prevent disease transmission in the pediatric population.

## 2. Materials and Methods

### 2.1. Study Design and Data Sources

A cross-sectional, descriptive, and exploratory study was designed to analyze the main genetic variability of SARS-CoV-2, by categorizing a total of 372,989 pediatric patients according to VOCs high predominance period [22,23,24]. The data for this study were collected from the compilation of SARS-CoV-2 sequences from Mexico. We performed a search in the GISAID database (https://www.gisaid.org/ (accessed on 20 October 2022)), with a submission deadline of 16 October 2022. The selection criteria included location (“North America/Mexico”), and a complete sequence length (≥29,000 kb). To exclude the possibility of another type of virus and host sequences, we included the specification of “hCoV-19” and “Human”, respectively. All sequences resulting from the filtering were downloaded with the patient status metadata [9]. The open-access dataset (https://datos.covid-19.conacyt.mx (accessed on 20 October 2022)) was used for pediatric clinical outcomes.

### 2.2. Variants of Concern High Predominance Estimation

The category of VOCs groups and single proteome amino acid (aa) changes were generated from the RNA sequences metadata. PANGO lineages were used to classify VOCs groups according to the WHO statements [25]. We used a Python programming script, with Pandas, Plotly, and NumPy libraries. The RNA sequences metadata processed the identification of the mutation name, frequency, single mutation variation, aa changes, and the structural protein genetic position. A series of data methodologies were involved in cleaning, normalizing, and standardizing variables. Afterward, timeline dynamics, stacked density, and other plots grouped by VOCs were constructed [23] (Figure S2). This methodology revealed the circulation pattern dynamics of the VOCs’ prevalence fluctuations over time.

Single aa mutations in all genetic sequences were sorted by frequency. Mutations occurring in more than 30% of all genetic sequences were classified as ‘high-frequency’. The objective was to investigate the distribution of these high-frequency mutations in pediatric patients in Mexico. To achieve this, a comparative analysis of prevalence was conducted between the pediatric and adult groups, using statistical tests.

Likewise, to spot variations within the distribution, the age variable was also statistically compared among the groups of the VOCs.

The VOCs’ high predominance was defined as a ≥50% proportion of a single variant type among all the sequences processed [26]. The high-predominancy distribution lineages were calculated weekly. Likewise, a geographical stratification was performed using the states as the defined area for regionalization. These results were used to classify pediatric cases according to the VOCs groups.

### 2.3. Pediatric Severity Indicators

Sociodemographic variables included age, gender, location (states), and ethnicity (self-referred as belonging to an ethnic group, indigenous group, and/or speaking an indigenous language). Clinical variables included clinical severity, such as ambulatory, hospitalization, and death classification. Then, we compare the COVID-19 pediatric severity indicators across the VOCs groups. Rates for hospitalization, mortality, and intensive care unit (ICU) admission, among others, were employed as epidemiological statistics. In addition, a geographical analysis of the distribution dynamics was also created.

### 2.4. Statistical Analysis

The continuous variables are presented by means and standard deviation (SD). Categorical variables were defined as frequency percentages (%) and associated using the Chi-square test. For continuous data, two-sided *p*-values were calculated using Kruskal–Wallis test and *t*-test. All *p*-values less than 0.05 were considered statistically significant. To estimate differential associations between the VOCs groups and pediatric clinical severity, binary logistic regression models were then fitted, with outcomes of (1) hospitalization, (2) ICU/AMV admission, and (3) death. Logistic regression using time-to-event Cox regression was processed, and the model was adjusted with the pediatric age category. The age ranges include infants (0–2 years), preschoolers (3–5 years), children (6–12 years), and adolescents (12–17 years). R software, version 4.1.1, was used for model generation and data analysis [27].

## 3. Results

### 3.1. SARS-CoV-2 Sequence Results in the General Population

A total of 75,348 complete SARS-CoV-2 genome sequences from Mexican patients were analyzed. Of these sequences, 52% (*n* = 37,839) corresponded to female patients and 47.7% (*n* = 34,610) to male patients. The mean age was 42.8 (±19.11) years old, with a 1-month to 105 year old range. Only 6.5% (*n* = 4965) corresponded to pediatric patients (<18 years old). Clinical outcomes were also reported as ambulatory patients in 41.1% (*n* = 30,981), hospitalized patients in 18.3% (*n* = 13,791), and death outcomes in 0.009% (*n* = 690). 

A total of 5,303,456 amino acid mutations were identified in comparison with the Wuhan original SARS-CoV-2 sequence [24]. The median frequency was 70.38 aa mutations per genome sequence. Genetic regions with higher mutation frequency were located in the Spike protein (S protein) and the non-structural proteins (NSP) with 50% (*n* = 2,651,728) and 33% (*n* = 1,750,140) mutations, respectively.

A total of twenty-five high-frequency mutations were identified recurrently across all the Mexican genetic sequences (Table 1). These high-frequency mutations were defined with a cut-of-point of 30% (*n* ≥ 22,604) prevalence of all the genome sequences. These mutations were found positioned mainly in the following proteins: Spike protein 61% (*n* = 16), Nucleocapsid protein 23% (*n* = 6), Membrane protein 7% (*n* = 2), and Envelope protein 3.78% (*n* = 1). Likewise, the occurrence and distribution of high-frequency mutations in the pediatric and adult sequences were also examined.

Interestingly, it was found that 22 of these high-frequency mutations had a higher prevalence in pediatric sequences compared to adults. Similarly, all these high-frequency mutations were also associated with the Omicron VOC lineage. To date, no reports on this matter have been documented. Therefore, it is worth mentioning that this result represents the first report of its kind, addressing the increased prevalence of these high-frequency mutations in pediatric sequences and their connection to the Omicron lineage. The substantial presence of these high-frequency mutations in the pediatric population holds significant implications, particularly concerning immunological and transmission features (Table S1).

A total of 77.8% (*n* = 59,784) of the genetic sequences were VOCs-related. Delta and Omicron lineages were the most frequent VOCs with 34% (*n* = 26,322) and 37% (*n* = 28,725), respectively. 

**Figure 1:**
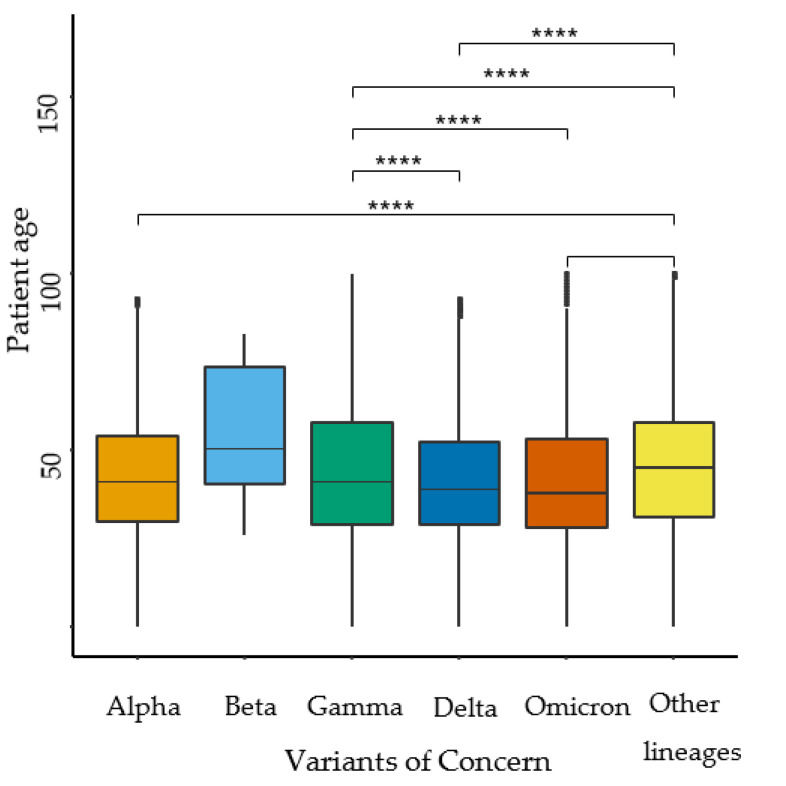
SARS-CoV-2 VOCs groups and age distribution comparison in Mexico (*n* = 75,348). Rank-Sum test **** (*p* < 0.0001).

To understand the significant increase in mutations related to the Omicron lineage observed in pediatric patients, an analysis was conducted to describe and compare the age distribution among different groups of VOCs (Figure 1). 

Interestingly, significant age distribution differences were found among the groups of VOCs (*p* < 0.001). It is worth noting that the mean age distribution for the Omicron lineage was 40.99 ± 18 years. This age distribution analysis in the VOCs provides valuable context and allows us to observe that the Omicron lineage exhibited a younger age distribution compared to Delta and other lineages (45 ± 19 years). This information could potentially help in understanding the transmission patterns and impacts on different age groups.

### 3.2. Variants of Concern High Predominance Circulation

**Figure 2:**
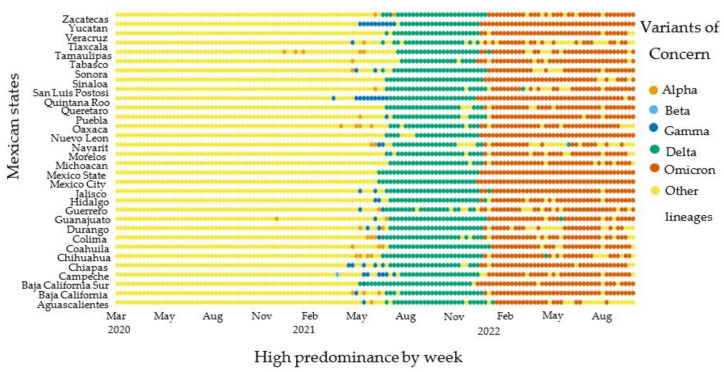
General population VOCs high-predominance circulation by week and geographic states (*n* = 75,348).

The high predominance model was calculated with a stratified method by analyzing total genetic sequences by week and the 32 federal entities (31 states and Mexico City). This methodology approach led to identifying differences in the VOCs circulation dynamics across the geographical regions in Mexico (Figure 2). Since 2021, we have been able to identify the origin of the VOCs circulation dominance dynamics in the country. The Delta VOC circulation started in Mexico City and Baja California state. Also, the Omicron circulation started in the states of Quintana Roo, Baja California, and Mexico City. 

A total of 372,989 COVID-19 confirmed pediatric patients were retrieved from SINAVE epidemiological platforms. A high-predominancy modeling distribution over the morbidity reported in pediatric patients was processed and classified. The age distribution among the pediatric population was 9% (*n* = 33,621) for infants (0–2 years), 8% (*n* = 32,669) for preschoolers (2–6 years), 35% (*n* = 130,949) for children (6–12 years), and finally 47% (*n* = 175,749) for adolescents (12–17 years).

**Figure 3:**
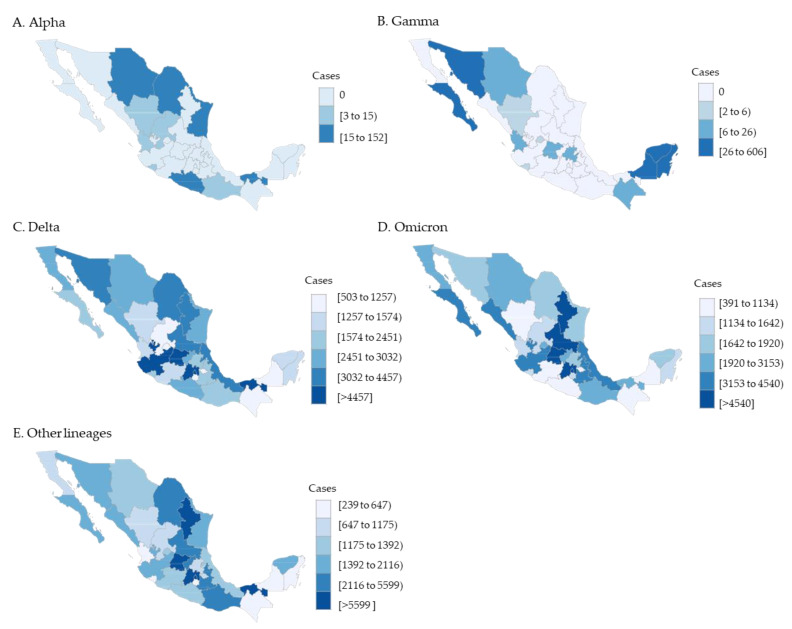
VOCs distribution in pediatric patients by geographic state (*n* = 372,989).

The geographical distribution of VOCs circulation among the COVID-19 pediatric cases showed regional differences. The pediatric Alpha lineages were congregated in Tamaulipas, Coahuila, Tabasco, and Chihuahua states. Gamma lineages in pediatrics also had an important outbreak restricted to the Yucatan peninsula region. This region includes Yucatan, Quintana Roo, and Campeche states. Delta, Omicron, and other lineages were consistently distributed in all Mexican federal entities, with an increased prevalence along the central region (Mexico City and the State of Mexico) (Figure 3).

### 3.3. Pediatric Severity Outcomes

A comparison of the principal pediatric severity outcomes across the VOCs groups is presented in Table 2. Sociodemographic factors such as age and sex were included. The age means distributions across the VOCs groups were similar. Only the Omicron lineage presented a decreased age mean of 10.33 ± 4.88 years, compared to other VOCs groups (*p* < 0.001). The male patients resulted in a slightly increased proportion versus the female COVID-19 patients. Only 1 to 4% of the pediatric patients were referred to as belonging to an ethnic group (indigenous group and/or speaking an indigenous language).

The clinical severity was classified as ambulatory, hospitalized, and death. Differences in the hospitalization proportions (*p* < 0.001) were estimated, with an increase of 8% in hospitalized patients in Alpha lineages. The death outcome was also increased in Alpha (1%) and the other lineages (1%) (Table 2).

Interestingly, a significant increase in automatized mechanical ventilation (AMV) and intensive unit care (ICU) requirements was attributed to Alpha (1%) and the other lineages (1%). Likewise, it was observed that the patients who most required ICU/AMV clinical support were in the infant group (0–2 years). This increased infant ICU/AMV use was found similarly in all groups of VOCs regardless of lineage (46–59%).

Pediatric hospitalization rates changed throughout all periods of the pandemic. For the other lineage group, the rate of pediatric hospitalization remained at 3.2 cases per week. Due to the introduction and widespread circulation of VOCs, there was a notable increase in hospitalization rates. Specifically, during the circulation of the Delta and Omicron VOCs, pediatric hospitalization rates reached 12.6 and 16.4 cases per week, respectively.

Significant differences were also found in the pediatric overall fatality rate, with an increase in pediatric cases affected by Alpha (0.4%) and the other lineages (0.7%), compared to the other groups of VOCs. Additionally, most pediatric mortality cases were reported within 28 days of clinical evolution (88–100%). These early mortality results remained consistent across all VOCs lineage groups, indicating a common trend. The high early mortality rate is likely attributable to COVID-19 severity during hospitalized admission and its subsequent associated complications.

Three logistic regression model analyses were conducted to compare hospitalization, ICU/AMV, and death pediatric outcomes, and to estimate the effect produced by VOCs lineages circulations in pediatric patients (Table 3). All three models resulted in significant statistics, and adequacy in the evaluation process (Figure S3). The hospitalization model showed an increase in the risk of hospitalization for the Gamma (IRR 2.67, CI [2.52–2.82]) and Alpha (IRR 1.69, CI [1.54–1.86]) pediatric patients compared to the other lineages. The ICU/AMV and death models also consistently showed this increase in the severity risk for the Gamma and Alpha VOCs compared to the other lineages. Likewise, the infant category resulted in an increased risk of hospitalization (IRR 1.27, CI [1.25–1.28]) compared to adolescents. Similarly, the infant category presented an increased risk of the ICU/AMV and death models. 

## 4. Discussion

This study discusses the severity of COVID-19 outcomes in Mexican pediatric patients and the most prevalent lineages affecting them. Therefore, we classified a total of 372,989 pediatric patients according to VOCs high predominance period. According to our findings, the most prevalent lineages among pediatric patients were the Omicron and Delta lineages. Alpha and the other lineages showed an increase in ICU/AMV admission and case fatality rates. A logistic model with age-adjusted variables estimated an increased risk of hospitalization, ICU/AMV, and death, in the Gamma and Alpha lineages compared to the other lineages. The use of this logistic regression model allowed us to have a more precise estimation of the risk for COVID-19 severity, while appropriately adjusting for potential age-related biases in the pediatric severity.

Single mutations, particularly in the Spike protein, have been found to improve transmission, virulence, and pathogenesis [6]. Our results reveal an increased frequency of Spike mutations in Mexican pediatric sequences. This observation aligns with previous studies, which also reported a high prevalence of Spike protein mutations linked to the Delta and Omicron lineages [28]. Spike protein mutations, particularly D614G, T478K, and P681H, have been shown to significantly increase SARS-CoV-2 virulence activity. These virulence factors contribute to clinical changes, such as an increased transmission of the virus, particularly at low viral loads, and an elevation in viral load within the upper airways (nose and trachea) [29].

Another characteristic among Mexican pediatric sequences was the increase in mutations associated with the Omicron lineage. Similarly, other authors in the USA observed an increase in pediatric cases during the circulation of Omicron [30,31]. In this study, the most important clinical pediatric implication is the increase in transmission and decrease in pathogenesis that is observed in the Omicron lineage compared to other variants [32].

Several research publications provided information on the characteristics of the spread of SARS-CoV-2 variants in Mexico [20,33]. However, it is important to note that the available studies are primarily focused on adult patients, and there appears to be a lack of investigation concerning the impact of these VOCs on pediatric patients. 

This study made a notable improvement by processing multiple stratifications per epidemiological week and geographical state, following Chintala et al.’s suggestion [34]. Thanks to this enhanced methodology, we identified periods of high predominance of both Alpha and Gamma VOCs in the Mexican pediatric population. Otherwise, only periods dominated by Delta, Omicron, and other lineages would have been detected. This advanced methodology significantly enriched the analysis, enabling the recognition of a wide range of VOCs circulating among pediatric patients.

An increase in pediatric hospitalization rates was documented during Delta and Omicron high prevalence periods. The COVID-19 hospitalization criteria include but are not limited to, respiratory distress, complications from underlying comorbidities, and social factors influencing health access. Lower hospitalization rates in the United States have been reported according to the CDC, USA [10,26,35]. Discrepancies around this increased hospitalization could be caused by the lack of vaccination in the Mexican pediatric population [17]. ICU/AMV severity and the case fatality rate also increased in Alpha and the other lineages in comparison to other VOCs groups. Comparably, other studies have found an association between the Alpha variant and an increase in hospitalization, ICU admission, and mortality [36,37]. Similarly, an increase in the clinical severity risk due to the Gamma and Alpha lineages was highlighted with a linear regression model. These age-adjusted models indicate a significant risk increase in hospitalization, ICU/AMV, and death compared to the other lineages. Additionally, studies have used these methods to estimate risk severity with similar results to our study [38]. In general, all VOCs showed a high early mortality rate (≥80%) in less than 28 days, which denotes that most of the deaths are directly attributed to the COVID-19 disease [39].

Another important feature was noted throughout the modeling estimates. An increase in severity risk was reported in infants (under 2 years). These patients presented a higher risk of suffering hospitalization, ICU/AMV, and death compared to adolescent patients. Likewise, studies on age-related clinical outcomes showed that children (under 5 years) were found to have greater complications and comorbidities throughout the COVID-19 pandemic [35,38]. 

This study also has implications for vaccination and complications in COVID-19 pediatric patients. Although it is very rare, some children can develop Multisystem Inflammatory Syndrome in Children (MIS-C) or become more likely to be newly diagnosed with diabetes after infection [40]. VOCs can cause severe illness and complications in children with underlying medical conditions or with special healthcare needs. In Mexico, the first COVID-19 vaccination was received for emergency use and authorization for ages 12 to 17 was given in September 2021 [17]. Vaccination for children aged 5 to 12 years started in March 2022. Unfortunately, the COVID-19 vaccination status in the pediatric population could not be determined because there is no proper record in the epidemiological surveillance systems (SINAVE) to be used for this analysis. However, it is important to note that vaccination for children under the age of 5 has not yet been approved in Mexico. Vaccination in the pediatric population will help the public health institutions to prevent COVID-19 and slow the spread of new lineages of SARS-CoV-2. 

### Study Limitations

The major strengths of this study include a large number of complete sequences and high representativeness of pediatric patients. As a limitation, GISAID sequences represent only a proportion of all COVID-19 cases. And the quality of the data depends on secondary databases. Further studies combining genomic variability, immunization status, comorbidities, epidemiological detail information, and clinical features of COVID-19 pediatric patients may be useful to characterize the physio pathological effects on the final pediatric clinical outcomes.

## 5. Conclusions

The VOCs have shown variations in circulation dynamics throughout time and geographical regions. According to a logistic regression analysis, periods of high predominance of Gamma and Alpha VOCs increased COVID-19 hospitalizations, ICU/AMV admissions, and mortality. Infant patients presented the worst clinical prognoses of severity in all types of VOCs. As a recommendation, we encourage childhood national immunization strategies and continued community- and government-based public health preventive interventions in elementary schools and daycare settings.

## Figures and Tables

**Table 1:** High-frequency single amino acid mutations associated with SARS-CoV-2 pediatric sequences (*n* = 75,348).

Gen/Mutation	Associated VOCs ^a^	Pediatrics		Adults		*p*-Value ^b^
		*n* = 4965	(%)	*n* = 71,873	(%)	
**Envelope**						
T9I	ο	2333	(46.8)	27,146	(37.7)	<0.001 ***
**Membrane**						
A63T	ο	2342	(47)	27,195	(37.8)	<0.001 ***
Q19E	ο	2251	(45.2)	26,235	(36.5)	<0.001 ***
**Nucleocapsid**						
G204R	α ɣ ο	2718	(54.6)	39,554	(55)	0.702
R203K	α ɣ ο	2716	(54.5)	39,650	(55.1)	0.534
P13L	ο	2346	(47.1)	27,374	(38)	<0.001 ***
E31del	ο	2293	(46)	26,228	(36.4)	<0.001 ***
R32del	ο	2293	(46)	26,235	(36.5)	<0.001 ***
S33del	ο	2266	(45.5)	25,892	(36)	<0.001 ***
**Spike**						
D614G	α β ɣ δ ο	4911	(98.7)	71,072	(98.8)	0.916
T478K	δ ο	3795	(76.2)	51,975	(72.3)	<0.001 ***
P681H	α ο	2774	(55.7)	38,490	(53.5)	<0.001 ***
G142D	ο	2558	(51.4)	33,179	(46.1)	<0.001***
H655Y	ɣ ο	2468	(49.6)	30,106	(41.8)	<0.001 ***
L452R	δ ο	2427	(48.7)	31,479	(43.7)	<0.001 ***
N679K	ο	2354	(47.3)	27,299	(37.9)	<0.001 ***
N969K	ο	2348	(47.1)	27,212	(37.8)	<0.001 ***
Q954H	ο	2346	(47.1)	27,212	(37.8)	<0.001 ***
D796Y	ο	2318	(46.5)	26,947	(37.4)	<0.001 ***
N764K	ο	2256	(45.3)	26,103	(36.3)	<0.001 ***
G339D	ο	2249	(45.2)	25,919	(36)	<0.001 ***
T95I	ο	2162	(43.4)	27,023	(37.5)	<0.001 ***
S375F	ο	2104	(42.2)	24,058	(33.4)	<0.001 ***
S373P	ο	2068	(41.5)	23,687	(32.9)	<0.001 ***
N501Y	α β ɣ ο	2013	(40.4)	26,458	(36.8)	<0.001 ***

^a^ GISAID SARS-CoV-2 complete genomes (2022). Variants of Concern: (α) Alpha, (β) Beta, (ɣ) Gamma, (δ) Delta, and (ο) Omicron. ^b^ Pearson’s Chi-square tests. *** (*p* < 0.001).

**Table 2:** Comparison of clinical severity and sociodemographic indicators by VOCs lineages in Mexican pediatric patients (*n* = 372,989).

	Alpha		Gamma		Delta		Omicron		Other Lineages		*p*-Value
	*n* Mean	% (SD)	*n* Mean	% (SD)	*n* Mean	% (SD)	*n* Mean	% (SD)	*n* Mean	% (SD)	
**Age**											<0.001 ^a^
Mean (SD)	11.83 (±5.07)		11.61 (±4.95)		11.19 (±4.88)		10.33 (±4.88)		11.45 (±5.0)		
**Sex**											<0.001 ^b^
Female	213	53%	551	47%	60,396	50%	77,291	49%	45,455	49%	
Male	191	47%	622	53%	60,403	50%	81,418	51%	46,399	51%	
**Social**											
Ethnicity	9	2%	47	4%	1662	1%	1275	1%	1004	1%	
Adolescent pregnancy	2	0%	5	0%	578	0%	531	0%	355	0%	
**Clinical outcomes**											<0.001 ^b^
Ambulatory	344	91%	1134	95%	116,139	96%	117,971	96%	89,517	94%	
Hospitalized	31	8%	52	4%	3978	3%	4532	4%	5,341	6%	
Death	3	1%	2	0%	321	0%	224	0%	654	1%	
**ICU/AMV**	4	1%	4	0%	368	0%	343	0%	1050	1%	<0.01 ^b^
Infants	0	0%	2	50%	162	44%	176	51%	618	59%	<0.001 ^b^
Preschoolers	0	0%	0	0%	24	7%	48	14%	66	6%	
Children	0	0%	1	25%	51	14%	46	13%	144	14%	
Adolescents	4	100%	1	25%	131	36%	73	21%	222	21%	
**7-Day Hospitalization Rate**	0.11	(±0.14)	0.29	(±0.44)	12.6	(±41)	16.4	(±44)	3.16	(±13)	<0.001 ^a^
**Case Fatality Rate**	3	0.495	2	0.170	330	0.273	240	0.151	650	0.707	<0.001 ^b^
**Early mortality rate (<28 days)**	3	100%	2	100%	283	88%	205	92%	605	93%	<0.001 ^b^

^a^ Two-sided *p*-values were calculated by Kruskal–Wallis test, or ^b^ Chi-square test for trend, as appropriate. Age range: infants (0–2 years), preschoolers (3–5 years), children (6–12 years), and adolescents (12–17 years). Alpha (*n* = 404), Gamma (*n* = 1173), Delta (*n* = 120,799), Omicron (*n* = 158,709), and Other lineages (*n* = 91,854).

**Table 3:** Multivariate logistic models of the pediatric severity outcomes (*n* = 372,989).

Model	Hospitalization			ICU/AMV			Deaths		
	IRR ^a^	95% CI	*p*-Value	IRR ^a^	95% CI	*p*-Value	IRR ^a^	95% CI	*p*-Value
**VOCs**									
Other lineages	—	—		—	—		—	—	
Alpha	1.69	1.54, 1.86	<0.001	1.67	1.52, 1.84	<0.001	1.68	1.52, 1.85	<0.001
Delta	0.97	0.96, 0.98	<0.001	0.99	0.98, 1.00	0.004	0.99	0.98, 1.00	0.049
Gamma	2.67	2.52, 2.82	<0.001	2.69	2.54, 2.85	<0.001	2.7	2.54, 2.85	<0.001
Omicron	0.95	0.94, 0.96	<0.001	0.97	0.96, 0.97	<0.001	0.97	0.96, 0.98	<0.001
**Age category**									
Adolescent	—	—		—	—		—	—	
Infant	1.27	1.25, 1.28	<0.001	1.12	1.10, 1.13	<0.001	1.10	1.09, 1.11	<0.001
Preschool	1.10	1.09, 1.11	<0.001	1.06	1.04, 1.07	<0.001	1.06	1.04, 1.07	<0.001
Child	1.03	1.02, 1.04	<0.001	1.02	1.02, 1.03	<0.001	1.02	1.02, 1.03	<0.001

^a^ IRR = Incidence Rate Ratio, CI = Confidence Interval, ICU/AMV= Intensive Care Unit admission or Automated Mechanical Ventilation.

## Data Availability

Additional data can be retrieved upon request. This project’s complete RNA sequences set are available at the GISAID EPI_SET ID: EPI_SET_230411vg (https://doi.org/10.55876/gis8.230411vg). For the Phyton code used in the analysis, a GitHub repository was created at (https://github.com/JesusColinV/Genetic_variability_Covid_MX (accessed on 3 February 2022)).

## Supplementary Materials

The following supporting information can be downloaded at: https://www.mdpi.com/article/10.3390/idr15050053/s1, Table S1: Viral activity effects reported in high-frequency mutations associated with pediatric sequences in Mexico [41,42,43,44,45,46,47,48,49,50,51,52,53,54,55,56,57,58]; Figure S1: A schematic illustration of the structural proteins of SARS-CoV-2. (A) S protein; (S1A) S1A domain, (S1B) S1B domain, (S1C) S1C Domain, (S1D) S1D domain, (PCS) protease cleavage site, (CβS) Central β-strand. (B) N protein; (NTD) N-Terminal, (RBD) Receptor Binding domain, (LINK) Predicted central linker, (DIM) Dimerization domain, (CTD) C-Terminal. (C) E protein; (NTD) N-Terminal, (TM) Transmembrane (CTD) C-Terminal. (D) M protein; (NTD) N-Terminal, (TMI) Transmembrane I domain, (TMII) Transmembrane II domain, (TMIII) Transmembrane III domain, (CTD) C-Terminal; Figure S2: Analysis of prevalence distribution across variants of Concern (VOCs) in Mexico (*n* = 75,348); Figure S3: Evaluation assessment plots for the clinical pediatric severity logistics models.
