# Supplementary material for: SARS-CoV-2 Variants of Concern and Clinical Severity in the Mexican Pediatric Population
Source: Infect Dis Rep. 2023 Sep 11;15(5):535–48. doi: 10.3390/idr15050053 (PMC10514801; doi:10.3390/idr15050053)
Supplement: Supplementary file 1 [file idr-15-00053-s001.zip › idr-2555364-supplementary.pdf]

infectious disease reports

MDPI

Article

SARS-CoV-2 Variants of Concern and Clinical Severity in the Mexican Pediatric Population

Anahí Maldonado-Cabrera [1,2,†], Jesus Alejandro Colin-Vilchis [3,†], Ubydul Haque [4,5], Carlos Velazquez [1], Andrea Socorro Alvarez Villaseñor [6], Luis Eduardo Magdaleno-Márquez [7], Carlos Iván Calleros-Muñoz [8], Karen Fernanda Figueroa-Enríquez [8], Aracely Angulo-Molina [1,9,*] and Ana Lucía Gallego-Hernández [1,*]

1 Department of Chemical Biological Sciences, University of Sonora, Hermosillo 83000, Mexico; a201203057@unison.mx (A.M.-C.); velaz2@unison.mx (C.V.)
2 Department of Epidemiology, Family Medicine Unit No. 37, Mexican Social Security Institute (IMSS), Hermosillo 83260, Mexico
3 Department of Medicine, Autonomous University of the Mexico State, Toluca 50000, Mexico; jcolinv859@alumno.uaemex.mx
4 Rutgers Global Health Institute, New Brunswick, NJ 08901, USA; uhaque@globalhealth.rutgers.edu
5 Department of Biostatistics and Epidemiology, School of Public Health, Rutgers University, Piscataway, NJ 08854, USA
6 Coordination of Medical Health Research, Mexican Social Security Institute (IMSS), La Paz 23920, Mexico; andrea.alvarez@imss.gob.mx
7 University Center for Biological and Agricultural Sciences, University of Guadalajara, Zapopan 44100, Mexico; eduardo.magdaleno@alumnos.udg.mx
8 Department of Agriculture, University of Sonora, Hermosillo 83323, Mexico; a219213704@unison.mx (C.I.C.-M.); a219218338@unison.mx (K.F.F.-E.)
9 School of Life Sciences, University of Applied Sciences and Arts Northwestern Switzerland, 4132 Muttenz, Switzerland
* Correspondence: aracely.angulomolina@fhnw.ch (A.A.-M.); ana.gallego@unison.mx (A.L.G.-H.)
† These authors contributed equally to this work.

Supplementary data

Table S1. Viral activity effects reported in high-frequency mutations associated with pediatric sequences in Mexico.

Gen/Mutation	Associated VOCs [a]	Effects in Viral Activity		
		Transmissibility	Immunity	Severity
**Envelope**				
T9I	o			Virulence decreased [41]
**Nucleocapsid**				
P13L	o		Decreased T cell recognition [42]	Decreased pathogenicity [43,44]
**Spike**				
T478K	δ o	Increase ACE2 affinity [45] Increased potential transmission [46]	Reduced affinity and antibody response [47,48] Increased antibody escape [45,49]	Increase virulence [45] Increased severity [50]
P681H	α o	Increase ACE2 binding [51] Increase infectivity and transmission [52]	Reduced affinity and antibody response [53,54]	Increased virulence [55] Increase replication, viral load [42]
G142D	o		Decrease antibody binding [41]	
H655Y	ɣ o	Increase ACE2 binding [51]		
L452R	δ o		Reduced affinity and antibody response [47]	
N679K	o	Increase ACE2 binding [51]		
N969K	o	Decrease infectivity [56]		
Q954H	o	Decrease infectivity [56]		
N764K	o			Increase virulence [57]
G339D	o		Increase antibody escape [58]	
S375F	o	Increased potential transmission [46]	Increase antibody escape [58]	
S373P	o	Increased potential transmission [46]	Increase antibody escape [58]	
N501Y	α β ɣ o	Increase infectivity and transmission [46,58]	Reduced affinity and antibody response [47,58]	

[a] Variants of Concern (α) Alpha, (β) Beta, (ɣ) Gamma, (δ) Delta, and (o) Omicron.

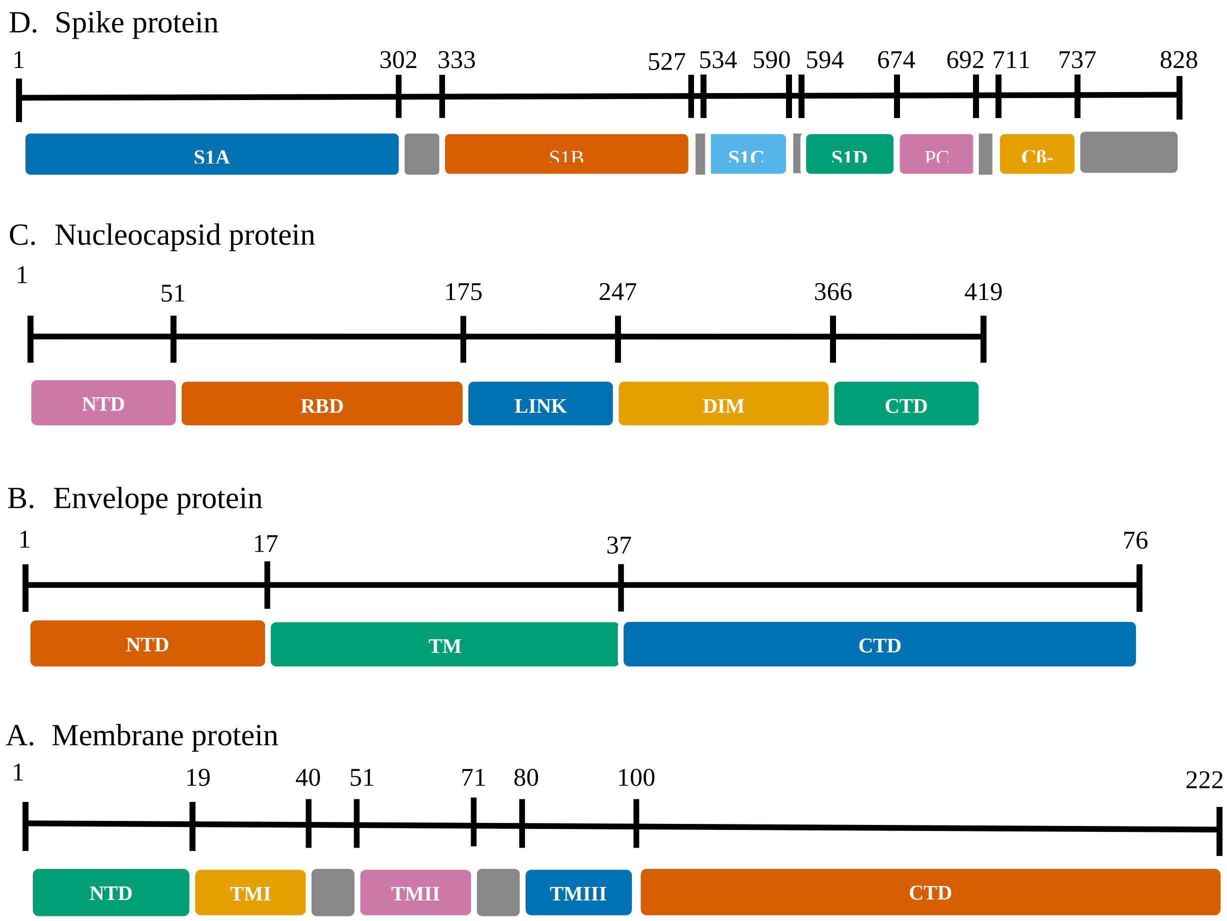

Figure S1. A schematic illustration of the structural proteins of SARS-CoV-2. (**A**) S protein; (S1A) S1A domain, (S1B) S1B domain, (S1C) S1C Domain, (S1D) S1D domain, (PCS) protease cleavage site, (CβS) Central β-strand. (**B**) N protein; (NTD) N-Terminal, (RBD) Receptor Binding domain, (LINK) Predicted central linker, (DIM) Dimerization domain, (CTD) C-Terminal. (**C**) E protein; (NTD) N-Terminal, (TM) Transmembrane (CTD) C-Terminal. (**D**) M protein; (NTD) N-Terminal, (TMI) Transmembrane I domain, (TMII) Transmembrane II domain, (TMIII) Transmembrane III domain, (CTD) C-Terminal.

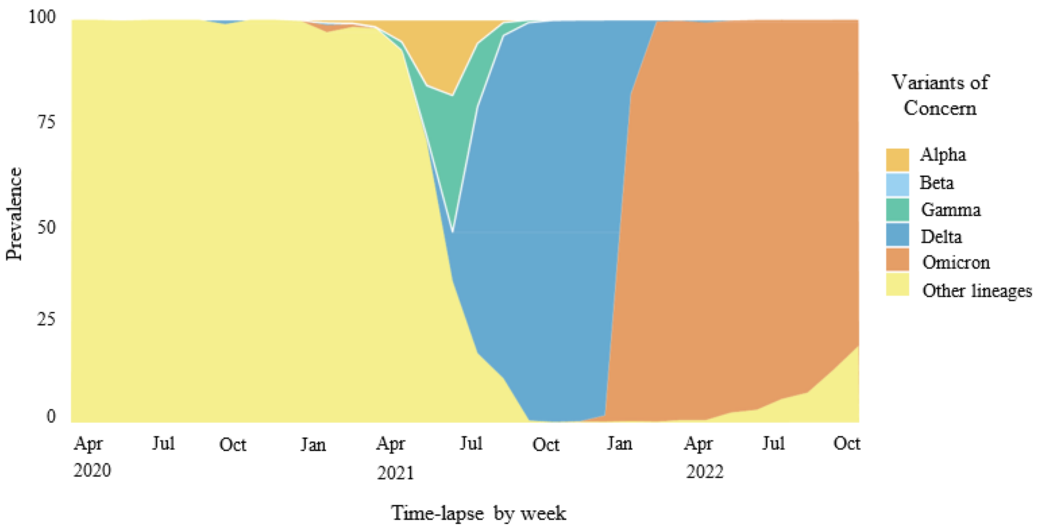

Figure S2. Analysis of prevalence distribution across Variants of concern (VOCs) in Mexico (*n* = 75,348).

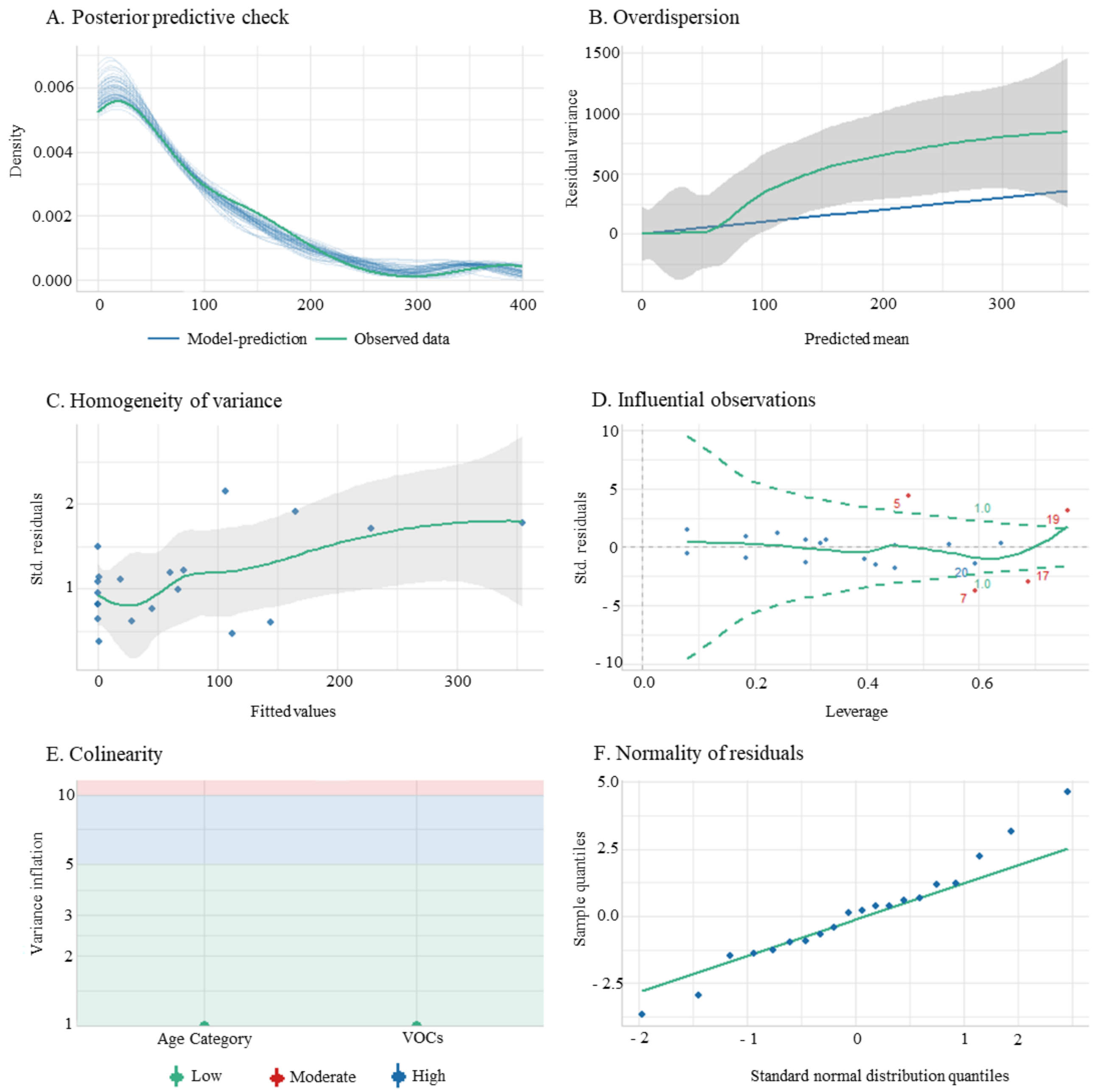

Figure S3. Evaluations assessments plots for the clinical pediatric severity logistics models. (**A**) Posterior predictive check; model-predicted lines should resemble observed data line. (**B**) Overdispersion and zero-inflation; observed residual variance (green line) should follow predicted residual variance (blue line). (**C**) Homogeneity of variance; reference line should be flat and horizontal. (D) Influential observations; points should be inside the contour lines. (E) Collinearity; High collinearity (VIF) may inflate parameter uncertainty. (F) Normality of residuals; dots should fall along the line.
